# Geographical Variation in Egg Rejection by Azure‐Winged Magpies (*Cyanopica cyanus*) Across China

**DOI:** 10.1002/ece3.71726

**Published:** 2025-07-07

**Authors:** Fudong Zhou, Yilin Lu, Jianping Liu, Wei Liang

**Affiliations:** ^1^ College of Biological Sciences and Engineering North Minzu University Yinchuan China; ^2^ Ministry of Education Key Laboratory for Ecology of Tropical Islands, Key Laboratory of Tropical Animal and Plant Ecology of Hainan Province College of Life Sciences, Hainan Normal University Haikou China

**Keywords:** azure‐winged magpie, brood parasitism, egg rejection behavior, geographical variation

## Abstract

Brood parasitism negatively affects the reproductive success of hosts, leading many hosts to evolve defense strategies to recognize and reject parasitized eggs. While studies have shown that hosts may adjust their defensive behavior according to parasitism risk, whether different geographical populations of the azure‐winged magpie ( *Cyanopica cyanus* ), which are parasitized by multiple cuckoo species and face varying parasitism risks, exhibit geographical variation in egg rejection behavior is unclear. As studies have shown that the color of model eggs can influence the egg rejection behavior of hosts, we also tested whether red or blue model eggs would affect the egg rejection behavior of the azure‐winged magpies. From April to June in 2023, we investigated the egg recognition ability of azure‐winged magpie populations in Fusong County, Jilin Province; Huangpi District, Hubei Province; and Luqu County, Gansu Province, China. The results showed that the azure‐winged magpie populations in all three regions rejected approximately 100% of the model eggs, with no significant difference in rejection rates between red and blue model eggs. This study revealed that Chinese populations of azure‐winged magpies exhibited high egg recognition ability. There was no geographical variation in their egg rejection behavior when presented with non‐mimetic blue or red model eggs. This study provided basic data for further research on the anti‐parasitic strategies of the azure‐winged magpie.

## Graphical abstract

Hosts may adjust their defensive behavior according to parasitism risk. Whether the azure‐winged magpie ( *Cyanopica cyanus* ) exhibits geographical variation in egg rejection behavior is unclear. Regardless of cuckoo parasitism status, all three azure‐winged magpie populations across China exhibited high recognition ability for non‐mimetic eggs, with no geographical variation in rejection behavior.

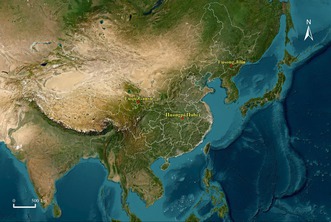

## 1 Introduction

Avian brood parasitism is a specialized reproductive strategy in which birds (such as cuckoos) lay eggs into host nests and rely on the host to provide parental care for their offspring (Davies 2000; Soler 2017). Brood parasitism imposes a high reproductive cost on host reproduction (Davies 2011; Soler 2014), prompting the evolution of various defensive strategies, such as selecting nest sites with lower parasitism risk, actively defending nests, and recognizing and rejecting foreign eggs or nestlings (Langmore et al. 2003; Expósito‐Granados et al. 2017; Tolvanen et al. 2017; Wang et al. 2023; Attisano et al. 2021; Ma and Liang 2021; Arco et al. 2023; Štětková et al. 2023; Zhang, Santema, et al. 2023). Among these, egg recognition and rejection are commonly used defense strategies (Davies 2011; Ye et al. 2022; Zhang, Zhong, et al. 2023; Yan and Liang 2024).

Brood parasitism is one of the strongest selective pressures driving the evolution of egg recognition in birds (Davies and Brooke 1989; Rothstein 1975, 1990; Ruiz‐Raya et al. 2016). However, additional factors such as nesting in dense colonies and nest usurpation may also contribute to the refinement of these discriminatory abilities (Peer and Bollinger 1998; Quach et al. 2021; Šulc et al. 2022; Alothyqi et al. 2024). Once a host species evolves egg recognition, this trait can be maintained at the population level over generations, even if brood parasitism temporarily ceases (Lahti 2006; Hale and Briskie 2007; Peer et al. 2011; Yang et al. 2014). However, some hosts gradually lose egg recognition when parasitism pressure is reduced (Thorogood and Davies 2013; Yang, Wang, et al. 2015). In addition, some studies have shown that host egg rejection behavior is individually repeatable, regardless of how the parasitic risk changes (Honza et al. 2007; Samaš et al. 2011; Grim et al. 2014), while some hosts will increase their rejection of previously accepted eggs when the perceived parasitic risk increases (Liu et al. 2021; Zhang et al. 2022; Li et al. 2025). Thus, studies on the effect of parasitism risk on host egg rejection behavior have produced inconsistent results.

The host–parasite interaction is spatiotemporally dynamic, with selective pressures varying according to the geographical distribution of hosts and parasites (Adamík et al. 2009; Møller et al. 2011; Yang et al. 2014). Even within a small geographical area, minor differences in nesting sites can result in variations in parasitism risk for a given host population (Begum et al. 2011; Lawson et al. 2023), and even more so for widely distributed species (Moskát et al. 2008; Liang et al. 2016; Yan and Liang 2024).

The azure‐winged magpie ( *Cyanopica cyanus* ) is widely distributed throughout the eastern Palearctic (Madge 2020; Madge and Juana 2020). In South Korea, although the azure‐winged magpie is predicted to be a potential host of the Indian cuckoo ( *Cuculus micropterus* ), parasitism has never been recorded (Lee 2014; Son et al. 2015). In Japan, azure‐winged magpies are not parasitized by the common cuckoo ( *Cuculus canorus* ) when their distributions are distinct; however, when their ranges coincide, they incrementally evolve into frequent hosts as cuckoos adapt to exploit this novel resource (Takasu et al. 1993). In China, azure‐winged magpies are distributed in many provinces, breeding from Heilongjiang Province in the north to Hainan Province in the south (Zheng 2023). The Indian cuckoo is distributed throughout the country (except Xinjiang region), and the Asian koel ( *Eudynamys scolopaceus* ) is distributed in the central and southern regions (Zheng 2023). In northern China, such as Shandong Province, Beijing, and the northern part of Henan Province, azure‐winged magpies have been found to be parasitized by the Indian cuckoo, while in central and southern China, such as Wuhan City in Hubei Province, they have been found to be parasitized by the Asian koel (Yang et al. 2012; Liu et al. 2025).

Owing to its wide distribution, azure‐winged magpies in different regions of China experience considerable variation in parasitism risk; however, whether they adjust their egg rejection behavior accordingly remains unclear. From April to August 2023, we studied the egg rejection behavior of azure‐winged magpies in Fusong County, Jilin Province; Luqu County, Gansu Province; and Wuhan City, Hubei Province, China. Considering that we had never found any cuckoos parasitizing azure‐winged magpies in their breeding sites in Fusong County and Luqu County, while in Wuhan City, azure‐winged magpies were parasitized by the Asian Koel, we hypothesized that azure‐winged magpies in Fusong and Luqu exhibit relatively low egg rejection rates; conversely, in Wuhan, they demonstrate significantly higher rejection rates.

## 2 Materials and Methods

### 2.1 Study Area

**FIGURE 1:**
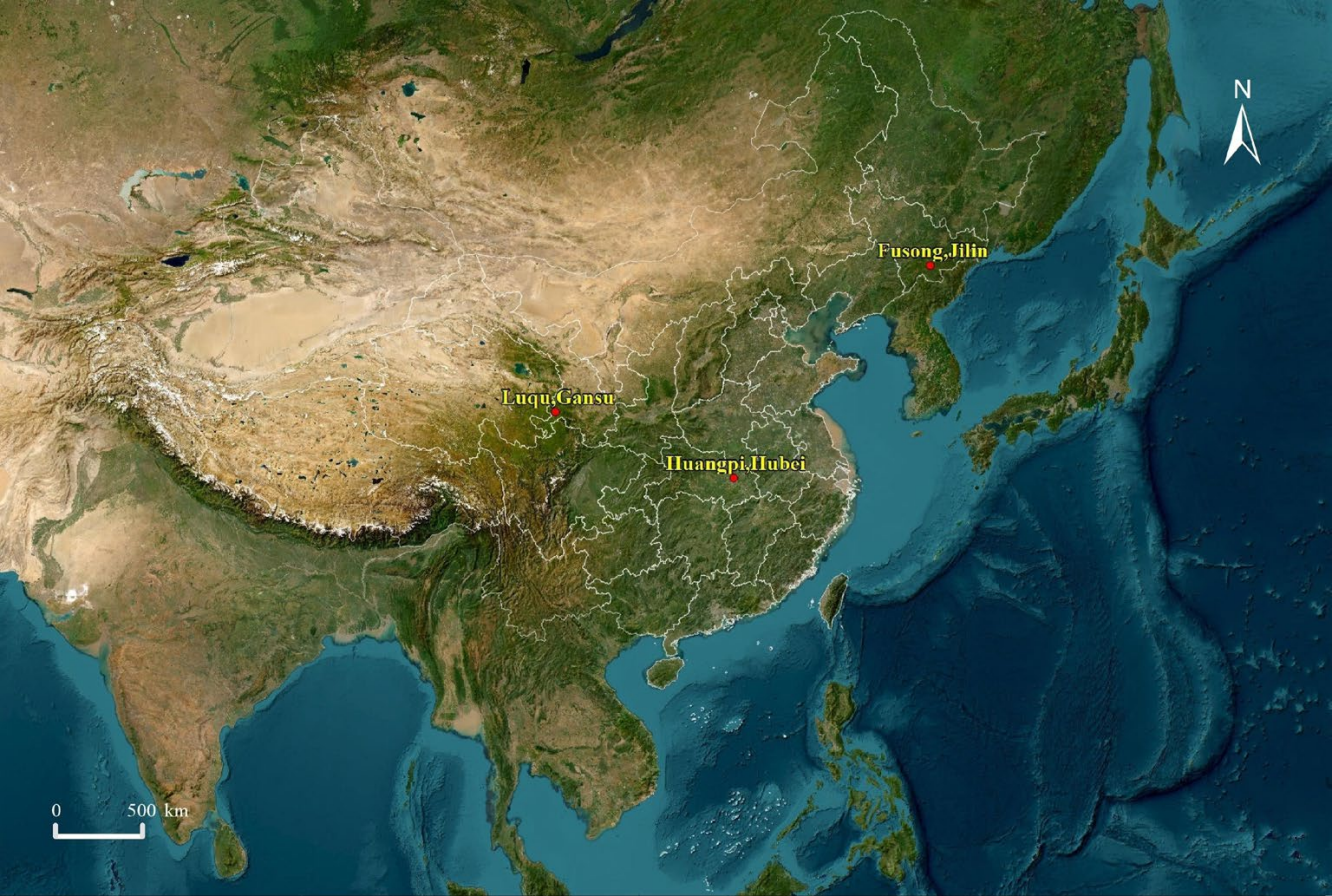
Locations of the three study sites across China.

Fusong County (42°19′ N, 127°15′ E) is located in Jilin Province, southeastern China (Figure 1) and is characterized by a continental monsoon climate. The dominant vegetation types are coniferous forests and mixed coniferous‐broadleaf forests (Liu, Wang, and Liang 2023). There are three species of cuckoos distributed in this area, including the common cuckoo, Indian cuckoo, and the lesser cuckoo ( *Cuculus poliocephalus* ) (Zheng 2023), and azure‐winged magpies are more likely to be parasitized by the Indian cuckoo. However, neither the historical documentary records nor our research observations at this area have found any evidence of azure‐winged magpies being parasitized. Huangpi District (30°42′ N, 114°10′ E) is an administrative district of Wuhan, situated in Hubei Province, central China (Figure 1). It is characterized by a subtropical monsoon climate, with dry and cold winters and hot, rainy summers. The dominant tree species include camphor ( *Cinnamomum camphora* ), the Chinese parasol tree ( *Firmiana platanifolia* ), and the golden rain tree ( *Koelreuteria paniculata* ) (Liu, Peng, et al. 2023). There are six species of cuckoos distributed in this area, including the common cuckoo, Indian cuckoo, Asian koel, lesser cuckoo, large hawk‐cuckoo (*Hierococcyx sparverioides*), and chestnut‐winged cuckoo ( *Clamator coromandus* ) (Zheng 2023). The azure‐winged magpies are more likely to be parasitized by both the Indian cuckoo and the Asian koel. Indeed, at this area, we only observed the Asian koel parasitizing the azure‐winged magpie. Luqu County (34°34′ N, 102°29′ E) is located on the eastern edge of the Qinghai‐Tibet Plateau in Gansu Province, southwestern China (Figure 1). It is characterized by a humid plateau climate, with cold and damp conditions. The primary vegetation types include meadows, coniferous forests, and shrublands (Gao et al. 2021). At this research site, we rarely observed any activity of cuckoos.

### 2.2 Egg Rejection Experiment

**FIGURE 2:**
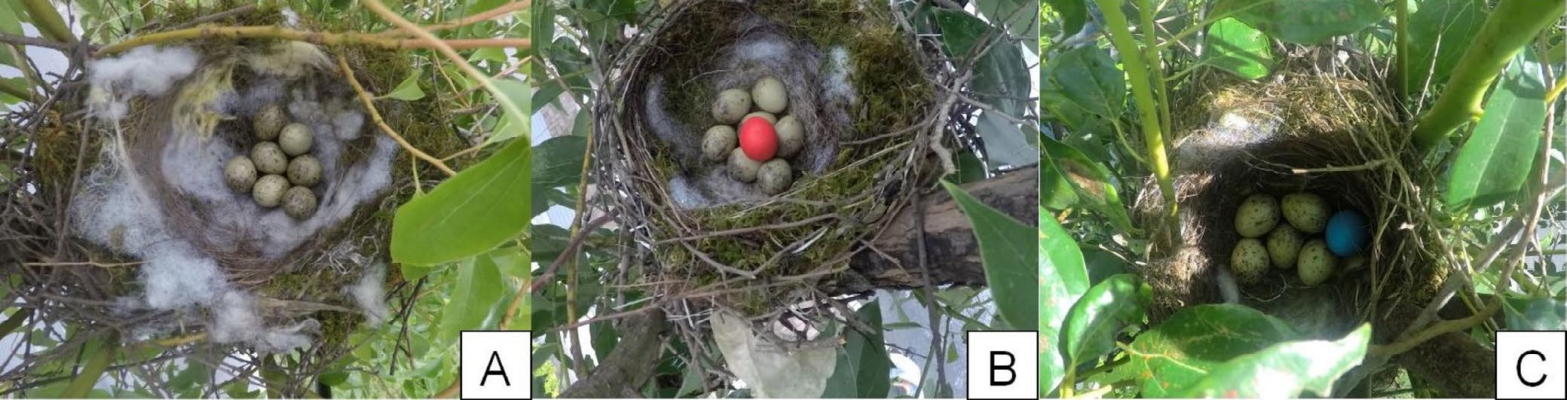
Nests, eggs, and model eggs used in the azure‐winged magpie experiments. A refers to the nest and eggs of azure‐winged magpies, B refers to the azure‐winged magpie nest with one added red model egg and C refers to the azure‐winged magpie nest with one added blue model egg.

During the azure‐winged magpie breeding season from April to June, 2023, we systematically searched for nests in the study area and recorded the nest coordinates and clutch size. Egg recognition experiments were conducted on nests in the early incubation stage (1 to 4 days after clutch completion). Since blue or red model eggs are widely employed in studies of host egg recognition as non‐mimetic stimuli (Rothstein 1990; Lahti 2006; Grim et al. 2011; Soler 2014; Samaš et al. 2014; Liu et al. 2024), and previous studies have suggested that the color of model eggs can influence hosts' rejection decisions (Abolins‐Abols et al. 2019; Hanley et al. 2021; Liu et al. 2024; Yan and Liang 2024), many cognitive models of foreign egg recognition have been built on the assumption that hosts use templates to compare their own eggs with potential foreign eggs to assess the similarity between host and parasite eggs (Stoddard and Stevens 2011; Hauber et al. 2015). The eggs that fall beyond a specific acceptance threshold are rejected (Reeve 1989). Therefore, we wondered whether non‐mimetic red and blue model eggs, which differ significantly from the eggs of the azure‐winged magpie, would affect the egg rejection decision of the azure‐winged magpie. To investigate the effects of color on the egg rejection behavior of the azure‐winged magpie, we tested the egg recognition abilities in all three study sites using both red and blue model eggs. The blue or red model eggs were both made of clay, with a size (long diameter of 26.05 ± 0.87 mm, short diameter of 20.07 ± 0.56 mm, weight of 5.71 ± 0.42 g, *n* = 30), and a shape similar to that of the magpie eggs (long diameter of 26.21 ± 1.05 mm, short diameter of 19.96 ± 0.89 mm, weight of 5.45 ± 0.75 g, *n* = 30), and slightly smaller than the Asian koel eggs (long diameter of 34.73 ± 0.74 mm, short diameter of 23.33 ± 0.12 mm, weight of 9.72 ± 0.21 g, *n* = 10). The experiment was divided into three groups: (1) no model egg was added, and the nest was monitored under natural conditions (Figure 2A); (2) one red model egg was added directly to the nest (Figure 2B) (Fusong, *n* = 16; Luqu, *n* = 11; Huangpi, *n* = 21); and (3) one blue model egg was added directly to the nest (Figure 2C) (Fusong, *n* = 16; Luqu, *n* = 22; Huangpi, *n* = 28). We observed the host's response to the experimental egg over 6 days. If the model egg remained in the nest on the sixth day without peck marks and the magpie continued normal incubation, it was classified as accepted. If the model egg was missing, it was classified as rejected. As in Moksnes et al. (1991), we excluded nests depredated during the experiment from the analysis.

### 2.3 Statistical Analyses

Statistical analyses were performed with IBM SPSS 20.0 (IBM Inc., Armonk, New York, USA) for Windows. We used a Generalized Linear Mixed Model (GLMM) to analyze the response (accepted or rejected) to different colored model eggs (blue vs. red), clutch size, and the experiment date when the model egg was added to the nest at different study sites (Fusong vs. Huangpi vs. Luqu). The response was the dependent variable, and the model egg color, study site, experiment date, and clutch size were independent variables. The nest ID was treated as a random effect. All tests were two‐tailed, and significance levels were all set at *p* < 0.05.

## 3 Results

In 2023, we found 112, 33, and 242 nests of the azure‐winged magpie in Fusong (Jilin), Luqu (Gansu), and Wuhan (Hubei), respectively. Cuckoo parasitism cases were not recorded in Fusong or Luqu. However, in Wuhan, 10 nests of the azure‐winged magpie were parasitized by the Asian koel, with a brood parasitism rate of 4.1%. No koel eggs were rejected by the magpies.

**FIGURE 3:**
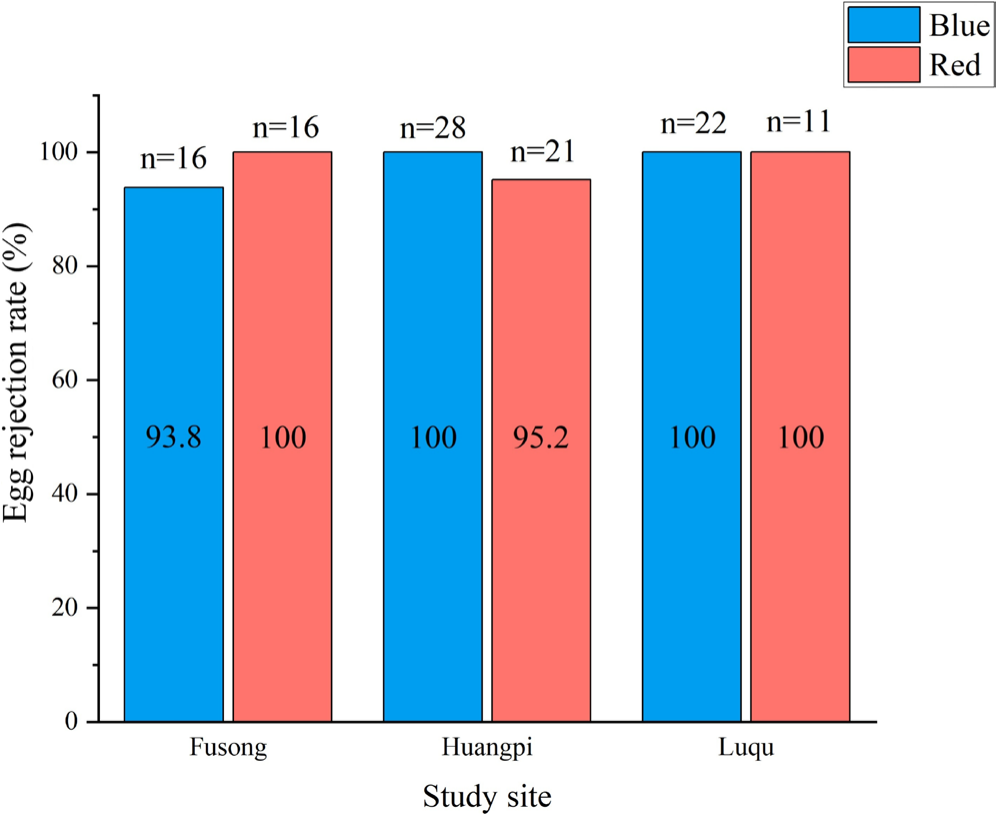
Egg rejection rates of azure‐winged magpie populations at the three study sites for blue and red model eggs.

GLMM analyses revealed no significant difference in the rejection rates of model eggs (*F* = 0.024, *p =* 0.98) or red and blue model eggs (*F* = 0.015, *p =* 0.90) by the azure‐winged magpies at the three sites (Figure 3). Additionally, neither the experimental date nor clutch size had a significant effect on the egg rejection responses of azure‐winged magpies (*p* > 0.05) (Table 1).

**TABLE 1:** Results of generalized linear mixed model analysis on the rejection rates of model egg colors by the azure‐winged magpies at the three sites.

Model	*F*	df_1_	df_2_	*p*
Site	0.024	2	103	0.976
Date	0.055	1	103	0.815
Clutch size	0.403	6	103	0.876
Model egg color	0.015	1	103	0.902

## 4 Discussion

Our study revealed no significant differences in egg recognition ability among the three azure‐winged magpie populations, all of which exhibited an exceptionally high rejection rate for non‐mimetic model eggs. In the Wuhan area, however, magpies did not reject Asian koel eggs, which were highly similar to their own eggs from a human perspective. Additionally, the magpies showed no preference between red and blue model eggs, and neither clutch size nor experimental date influenced their egg rejection behavior.

Geographical differences in the rejection rates of non‐mimetic eggs by azure‐winged magpies were not observed, which was not consistent with our predictions. Generally, spatially separated host populations exhibit different responses to parasitic eggs owing to differences in environmental conditions, life‐history strategies, and parasitism risk (Soler et al. 1998). Several studies support this phenomenon. For example, Yang, Hu, et al. (2015) compared the egg recognition ability of European and Asian house sparrow ( *Passer domesticus* ) populations and found that house sparrow populations in Asia lacked egg recognition ability, whereas European house sparrows exhibited strong egg rejection. Liang et al. (2016) examined the egg rejection behavior of several populations of great tits ( *Parus major* ) in Europe and China and found that European populations had little egg recognition ability, whereas Chinese populations exhibited very high rejection rates with a latitudinal gradient, declining from 100% in the south to 52% in the north. These differences may be related to parasitism risk. In most of Europe, only the common cuckoo parasitizes hosts, and its large body size likely prevents it from laying eggs in great tit nests, limiting coevolutionary interactions. However, China has many cuckoo species, and some smaller‐sized cuckoos may enter great tit nests to lay eggs and engage in parasitism (Yang et al. 2012).

Our research results are different from those of previous studies. Across all three study sites, azure‐winged magpies demonstrated high egg recognition ability, with rejection rates of approximately 100%. Although cuckoo parasitism was not observed in Fusong (Jilin), the limited one‐year observation period precludes definitively ruling out its occurrence in these regions. In Fusong, near our research site, the calls of the Indian cuckoo could often be heard, which also suggested that the azure‐winged magpies might still be at risk of being parasitized by the Indian cuckoos. After all, azure‐winged magpies were found to be parasitized by Indian cuckoos in another population (Benxi City, Liaoning Province) 320 km away from Fusong (Liu, Wang, and Liang 2023). Therefore, the rejection rate of model eggs by magpies at this study area was relatively high. The azure‐winged magpie population in Luqu, although no cases of brood parasitism by cuckoos have occurred, its distribution has gradually expanded to the Tibetan Plateau from lower latitudes in the past few decades (Ren et al. 2016; Gao et al. 2020). The high rejection rate of model eggs by this population might be due to the interaction between this species and cuckoos in the lower latitudes. Furthermore, the azure‐winged magpies in Fusong and Luqu might simply be former hosts that have successfully escaped parasitism by evolving excellent discrimination abilities. And these azure‐winged magpies maintain a high egg rejection response when not parasitized. Studies on the red‐billed leiothrix ( *Leiothrix lutea* ), song thrush ( *Turdus philomelos* ), blackbird ( *Turdus merula* ), chaffinch ( *Fringilla coelebs* ), and Bohemian waxwings ( *Bombycilla garrulus* ) revealed that hosts exhibit extremely high rates of egg rejection in the absence of parasitic risk (Hale and Briskie 2007; Peer et al. 2011; Yang et al. 2014). In addition, Korean populations of azure‐winged magpies, which are also not parasitized by cuckoos, maintain the same high egg rejection behavior (Son et al. 2015).

Visual cues are crucial for many bird hosts in recognizing and rejecting foreign eggs (Ruiz‐Raya and Soler 2020; Samaš et al. 2021). Hosts can reject foreign eggs by directly comparing the phenotypic differences between foreign and their own eggs in the nest (Antonov et al. 2006; Štětková et al. 2023). Furthermore, blue or red model eggs are widely employed in studies of host egg recognition as non‐mimetic stimuli (Rothstein 1990; Lahti 2006; Grim et al. 2011; Soler 2014; Samaš et al. 2014; Liu et al. 2024). Some hosts exhibit lower rejection rates toward specific non‐mimetic colors, suggesting either sensory biases in their recognition system or evolved tolerance thresholds. For example, bush warblers ( *Cettia diphone* ), green‐backed tits ( *Parus monticolus* ), and barn swallows ( *Hirundo rustica* ) exhibit lower rejection rates toward brown or reddish colored foreign eggs (Higuchi 1989; Liu et al. 2024; Yan and Liang 2024). However, blackbirds, American robins ( *Turdus migratorius* ), chalk‐browed mockingbirds ( *Mimus saturninus* ), and common redstarts ( *Phoenicurus phoenicurus* ) are more likely to reject brown eggs while accepting blue ones (Hanley et al. 2017, 2019; Manna et al. 2020). However, our study revealed no significant differences in rejection rates between the two colors of non‐mimetic eggs in azure‐winged magpies, as rejection rates for red and blue eggs were similarly high. The multiple rejection threshold model posits that hosts have an acceptance range for the absolute perceivable differences between foreign eggs and their own egg coloration. Stimuli falling between the minimum and maximum thresholds are accepted, whereas those exceeding the thresholds are rejected (Hanley et al. 2017, 2019). Although we did not measure the egg spectral properties, our findings align with the “multiple rejection threshold model”. The background color of azure‐winged magpie eggshells is typically gray‐white or gray‐green, and both the short‐wavelength blue model eggs and long‐wavelength red model eggs may fall outside their acceptance threshold, leading to the rejection of both (Hanley et al. 2019).

In conclusion, although the blue eggs might not represent a “real” parasitism situation, our study demonstrated that regardless of cuckoo parasitism status, all three azure‐winged magpie populations across China exhibited high recognition ability for non‐mimetic eggs, with no geographical variation in rejection behavior toward non‐mimetic eggs. However, this uniformity does not necessarily imply an absence of variation in their rejection behavior toward more mimetic eggs. Moreover, a preference between red and blue model eggs was not exhibited. Further research is needed to determine whether egg rejection behavior in the azure‐winged magpie is likely to have a genetic component, or social learning can enable the rapid dissemination of rejection behavior throughout a host population. Marking the azure‐winged magpie with leg bands and manipulating the social information available from a neighboring nest might help to solve these problems.

## Author Contributions

**Fudong Zhou:** data curation (equal), formal analysis (equal), investigation (equal), methodology (equal), writing – original draft (equal). **Yilin Lu:** investigation (equal), methodology (equal). **Jianping Liu:** conceptualization (equal), formal analysis (equal), funding acquisition (equal), resources (equal), supervision (equal), validation (equal), writing – review and editing (equal). **Wei Liang:** conceptualization (equal), funding acquisition (equal), supervision (equal), writing – review and editing (equal).

## Ethics Statement

The experiments comply with the current laws of China, where they were performed. Experimental procedures were in agreement with the Animal Research Ethics Committee of Hainan Provincial Education Centre for Ecology and Environment, Hainan Normal University (No. HNECEE‐2014‐005).

## Conflicts of Interest

The authors declare no conflicts of interest.

## Supporting information


Data S1.

## Data Availability

Data used for this study are provided as Table S1 and can be found at https://figshare.com/s/a376ea2ba128ccb8e892 (doi: 10.6084/m9.figshare.28748627).
